# Aged rats learn Morris Water maze using non-spatial search strategies evidenced by a parameter-based algorithm

**DOI:** 10.1515/tnsci-2022-0221

**Published:** 2022-07-01

**Authors:** Eliud Enrique Villarreal-Silva, Alejandro Rafael González-Navarro, Rodolfo Amador Salazar-Ybarra, Oscar Quiroga-García, Miguel Angel de Jesús Cruz-Elizondo, Aracely García-García, Humberto Rodríguez-Rocha, Jesús Alberto Morales-Gómez, Alejandro Quiroga-Garza, Rodrigo Enrique Elizondo-Omaña, Ángel Raymundo Martínez-Ponce de León, Santos Guzmán-López

**Affiliations:** Neurosurgery and Neuroendovascular Therapy Department, “Dr. José Eleuterio González” University Hospital, Universidad Autónoma de Nuevo León, Madero Av. and Dr. Aguirre Pequeño s/n. Col. Mitras Centro, Monterrey C.P. 64460, Nuevo León, México; Human Anatomy Department, Universidad Autónoma de Nuevo León, School of Medicine, Nuevo León, México; Histology Department, Universidad Autónoma de Nuevo León, School of Medicine, Nuevo León, México

**Keywords:** Morris water maze, search strategy, aging, learning, memory, hippocampus

## Abstract

Spatial learning and memory are used by all individuals who need to move in a space. Morris water maze (MWM) is an accepted method for its evaluation in murine models and has many protocols, ranging from the classic parameters of latency, distance, and number of crossings to the platform zone, to other more complex methods involving computerized trajectory analysis. Algorithm-based SS analysis is an alternative that enriches traditional classic parameters. We developed a non-computerized parameter-based Search Strategy Algorithm (SSA), to classify strategies and detect changes in spatial memory and learning. For this, our algorithm was validated using young and aged rats, evaluated by two observers who classified the trajectories of the rats based on the effectiveness, localization, and precision to reach the platform. SSA is classified into 10 categories, classified by effectiveness, initial direction, and precision. Traditional measurements were unable to show significant differences in the learning process. However, significant differences were identified in SSA. Young rats used a direct search strategy (SS), while aged rats preferred indirect ones. The number of platform crossings was the only variable to show the difference in the intermediate probe trial. The parameter-based algorithm represents an alternative to the computerized SS methods to analyze the spatial memory and learning process in young and age rats. We validate the use of SSA as an alternative to computerized SS analysis spatial learning acquisition. We demonstrated that aged rats had the ability to learn spatial memory tasks using different search strategies. The use of SSA resulted in a reliable and reproducible method to analyze MWM protocols.

## 1 Introduction

Aging is a universal process in which we accumulate irreversible, time-dependent changes that predispose the individual to illness and inevitably lead to death [1]. Murine models are used to study normal and pathological aging to understand the neurological changes related to age [2,3,4,5,6,7]. Spatial memory works as a bridge from which memory mechanisms can be studied in aging neuroscience, due to the conservation of mechanisms to acquire spatial memory among different species [8]. Radial Mazes, T-mazes, and Water mazes are some of the most accepted methods to evaluate spatial learning and memory acquisition in small rodents.

Morris water maze (MWM) is used to assess hippocampal functions, aging, and neurogenesis among others [3,9,10,11]. Several studies have reported that MWM performance declines heterogeneously along with lifespan [12]. Physical and physiological changes such as weight, stress levels, and sensorial changes related to age could affect this performance; also, cognitive changes are involved in this restricted capacity, which reflects the decreased hippocampal neurogenesis levels in aged rats [10,13,14]. Although the study of aging and hippocampal functions has a wide variety of methods, such as molecular markers, mostly related to oxidative stress and hypoxia [13,15]; functional analyses that differentiate performance between young and aged rats are needed.

Traditionally, MWM consists of acquisition and probe trials. In the acquisition trail, latency and distance traveled until the platform is found are the most used variables, while in the probe trial the time spent in the correct quadrant and the number of crossings on the platform zone are used [16]. An alternative to traditional measurements is the analysis of the behavior described as a search strategy (SS) which refers to the sequence of movements the rodent use until the platform is found [17].

The behavioral analysis assesses the use of cognitive skills, giving a secondary role to the physical changes that affect the aged animals. Computational analysis of MWM has been widely described [18,19,20]; however, a manual parameter-based analysis could be used as a chain between traditional measurements and highly complex methods, primarily in young research groups. The aim of our study is to compare the MWM performance of aged versus young rats using traditional measurements and SS analysis using a parameter-based algorithm.

## 2 Methods

We performed a prospective, analytical, experimental study in the Universidad Autónoma de Nuevo León using Wistar rats. The project was previously submitted and approved by the University’s Ethics and Research Committees under registration number AH11-001. Animal care was performed in accordance with the criteria established by the institutional laboratory animal care and use committee, as well as federal regulation NOM-062-ZOO-1999.

### 2.1 Animals

Sixteen male Wistar rats were used in this experiment, eight young (3–6 months) and eight aged (18–21 months). All animals were housed in clear polycarbonate cages (two rats per cage) under 12-hour light-dark cycles, with access to food and water *ad libitum*. Temperature (22 ± 2°C) and humidity (60%) were kept constant. Animals with a bad general health status after or during the experimental analysis were excluded. After the second probe trial, three aged rats were excluded because of continuous thigmotaxis and feeding problems; these rats were substituted with three healthy aged Wistar rats.

### 2.2 Morris water maze protocol

**Figure 1:**
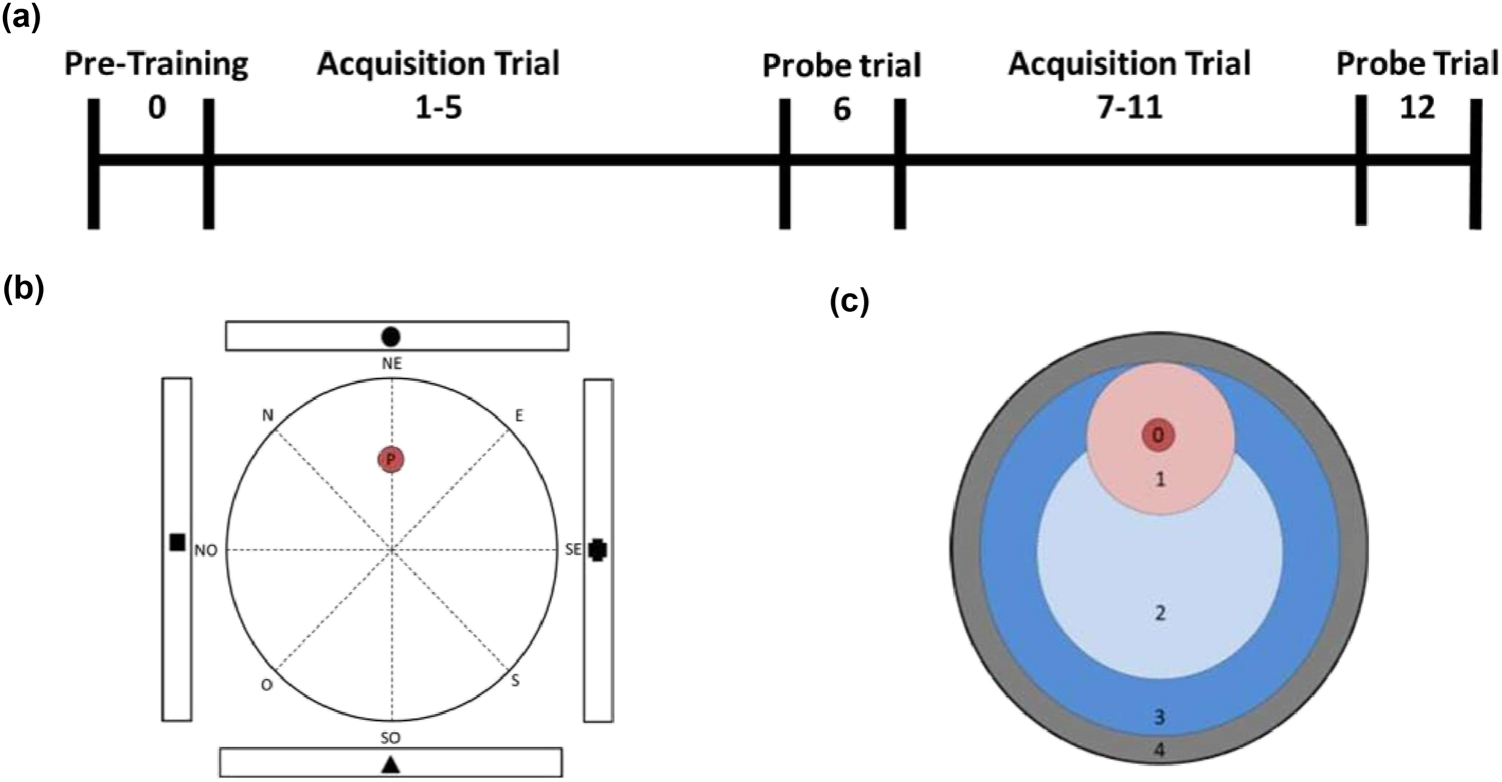
MWM protocol description: (a) Three different protocols were performed during the experiment: Pre-training, acquisition, and probe protocol. (b) MWM layout with figures in the extremes of each axis; dotted lines are imaginary axes. N: north, E: east, S: south, W: west. (c) MWM zones for SS analysis. Zone 0: platform zone, zone 1: peripheric platform zone, zone 2: the center of the pool zone, zone 3: the intermediate zone, and zone 4: the outer area of the pool.

We analyzed the MWM performance in aged and young rats using traditional measurements and the SS analysis during learning and probe trials. Young and aged rats were tested in MWM for 12 consecutive days (Figure 1a). The water maze consists of a round pool, 180 cm in diameter and 60 cm deep, filled with water (23 ± 2°C) made opaque with black non-toxic tempera paint. The pool was surrounded on each side by four white curtains with black figures used as cues (circle, triangle, square, and cross). To allow better identification of the seeking zones and SS, the pool was imaginarily divided in octants by eight imaginary axes (N, S, E, W, NE, NW, SE, and SW) (Figure 1b); a 15 cm diameter escape platform was hidden 2 cm below the water surface in a fixed location (NE axis) during all acquisition trial, halfway between the wall and the center of the pool. Additionally, to allow the identification of initial SS directionality, the pool was imaginarily divided into five circular zones to further examine the SS during the evaluations (Figure 1c). Zone 0 was the platform zone, zone 1 was the peripherical platform zone (75 cm diameter), zone 2 is the central pool zone (90 cm diameter), zone 3 is the peripherical central pool zone (150 cm diameter), and zone 4 represents the thigmotaxis zone (180 cm diameter).

Three different trials were performed during the experiment: pre-training, acquisition, and memory (Figure 1a). (1) *Pre-training trial.* Performed on day 0 to habituate the rats to the pool and water. It consisted of four trials of 90 s each, in which the rat must swim around in the pool and arrive at a visible central platform without the use of the curtains with cues. (2) *Acquisition trial.* Performed on days 1 to 5 and 7 to 11 after the pre-training to allow the rat to learn the fixed localization of the non-visible platform. It consisted of four trials per day (90 s each, with inter-trial intervals of 5 min), in which the rat began each trial from three different pseudo-random starting points and must guide its search using the peripheral cues. If a rat did not find the platform, it was set on it at the end of the trial for 30 s to allow the visualization of the cues. (3) *Probe trial.* Performed on the 6th and 12th days to assess the acquisition of memory. The platform was removed from the pool. It consisted of a 90 s trial in which the rats must identify the zone from which the platform was removed.

### 2.3 Assessment method

#### 2.3.1 Traditional measurements

All the traditional measurements and video tracks were performed with a computerized tracking system (ANY-maze^®^; Wood Dale, IL, USA). In the pre-training trial, the arrival to the visible central platform and swim velocity were quantified. In the acquisition trial, the platform latency (total time spent to reach the platform) and platform distance (total length of the swim path until reaching the platform) were quantified. In the probe trial, the correct quadrant time (percentage of time in the correct quadrant) and the platform zone crossings (number of crossings to the platform zone) were evaluated as well. The platform latency and distance until the first entry to the platform zone (zone 0) were also evaluated in the acquisition and probe trials.

#### 2.3.2 Search strategy analysis

**Figure 2:**
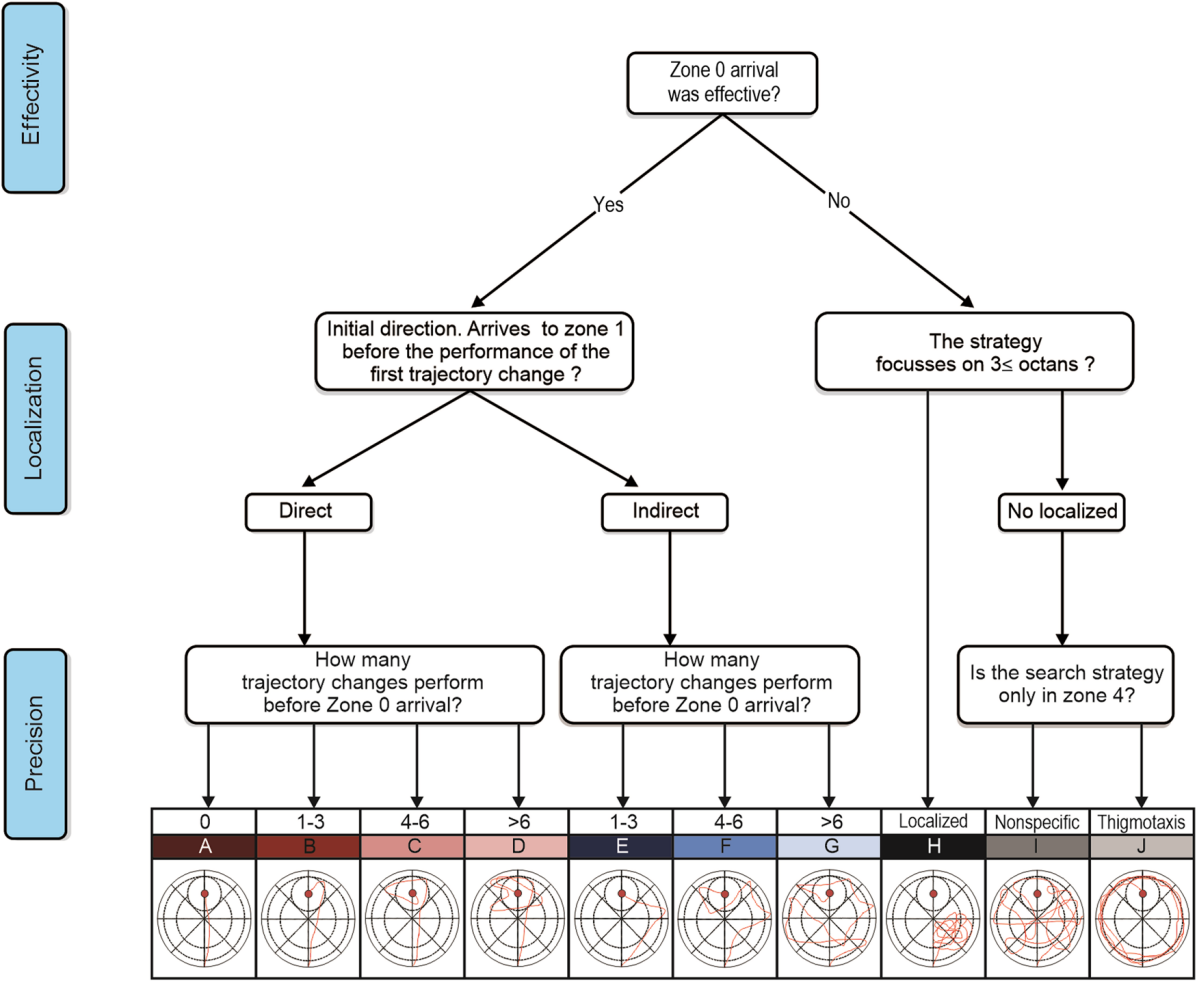
SSA: Search strategies can be classified objectively by the observer after specifying (a) effectivity, (b) initial directionality, and (c) precision. Direct strategies are classified under red colors; indirect strategies under blue colors; nonlocalized and ineffective strategies as black-grey colors.

The SS analysis in the acquisition and probe trials was performed using a parameter-based algorithm (Figure 2), with ten possible categories. Two independent observers were blinded to the group distribution and analyzed manually the SS using the video tracks. A third independent observer assessed the classification in the case of any discrepancies between the first two observers.

The algorithm initially classifies effectivity. If the rat arrives at the platform zone (zone 0), it is determined as an effective SS (red and blue categories: A to G). If the rat does not arrive at the platform, it is ineffective (black-grey colors: H, I, and J). Ineffective search strategies were then classified as follows: J-Thigmotaxis if the seeking was limited to the peripherical pool zone (zone 4), I-Ineffective and nonlocalized if the seeking was random in the pool, and H-Ineffective and localized if the seeking was limited in three contiguous octants. Effective search strategies were then classified using initial directionality.

**Figure 3:**
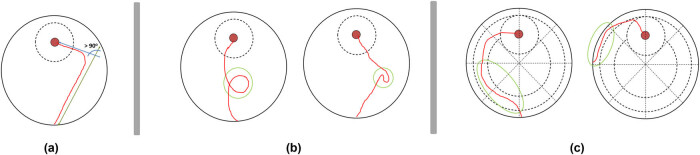
Trajectory changes – the SSA identified three different types of trajectory changes: (a) curve trajectory, (b) non-sense movements, and (c) peripheric trajectories.

Initial directionality was evaluated by observing the moment of the first trajectory change. A trajectory change is considered a mistake during SS. The possible trajectory changes are illustrated in Figure 3 and were defined as one the following: *curved movement* (with more than 90⁰ of angulation), *nonsense movements* (zigzag or spiral movements), and *peripheral seeking* (crossing more than three contiguous octants in zone 3 or one octant in zone 4. Considering this, initial directionality was classified as a direct SS (red colors: A to D) if the rat arrives at the peripheral platform zone (zone 1) before the performance of the first trajectory change and an indirect SS (blue colors: E to G) if the rat performs the first trajectory change before arriving the peripheral platform zone. After initial directionality classification, the precision was classified.

Finally, the precision classification was performed considering the number of trajectory changes until arriving at the platform zone (zone 0). Among effective and direct (red colors), it was classified as an A if zero trajectory changes, B if 1 to 3, C if 4 to 6, and D if ≥7. Among effective and indirect (blue colors), it was classified as E if 1–3 trajectory changes, F if 4–6, and G if ≥7.

With these three parameters: effectivity, initial directionality, and precision, the search strategies can be ordinally classified. As a theoretical example, in the initial probe trials, a rat must progress from thigmotaxis (J) to nonspecific (I-ineffective, non-localized, and imprecise) and then to an infective but localized (H). With continuous probe trials, the rat starts to identify the platform (zone 0) making their strategies effective but indirect (blue colors). The rat will diminish the number of trajectory changes until arrives at the platform zone making it more precise and direct. At the end of the probe trials, the rat must arrive at the peripherical platform zone (zone 1) before the performance of the first trajectory change; after this, the rat will progressively diminish the number of trajectory changes until arriving at the platform zone (zone 0), making it more and more precise ideally reaching an effective, direct, and precise SS (A).

Considering this, the algorithm allows the distinction between effective and direct (red colors: A, B, C, and D), effective and indirect (blue colors: E, F, and G), and ineffective and imprecise or impaired search strategies (grey and black colors: H, I, and J) (Figure 2). All observers were trained and qualified using these instruments for SSA classification, prior to experiment assessment.

### 2.4 Statistical analyses

All statistical analysis was performed with the SPSS Version 24.0 (IBM Co., Armonk, NY, USA). Graphical data were made with GraphPad PRISM software version 8.0 (GraphPad Software, San Diego, CA, USA).

For statistical analysis, the independent variable was the rat’s age and the dependent variables were traditional variables and the SS. For traditional variables, normality test distribution was determined using the Kolmogorov–Smirnov test. Non-normal data were expressed as the median and interquartile range (IQ). Mann–Whitney U test and Friedman with Bonferroni post-hoc correction test were used for group comparisons. For SS variables, qualitative categorical data were pooled in sets of direct (A–D SS, Figure 1), indirect (E–G SS, Figure 1), and non-effective (H–J SS, Figure 1) for its analysis. These data are reported as frequency distributions, and comparisons were made using the chi-square test. Comparisons between inter-observer and intra-observer data were assessed using the Kappa index. *p*-Value less than 0.05 (*p* <  0.05) was considered statistically significant.

## 3 Results

### 3.1 Traditional measurements in the acquisition and probe trials

The speed swimming velocity was evaluated finding a lower swimming velocity in the aged rats. 19.9 cm/s (IntQR, 16.3–21.7 cm/s) for the aged and 20.3 cm/s (IntQR, 15.7–25.5 cm/s) for young. Mann–Whitney U test showed a significant difference in this parameter (*p* <0.001). These changes could be attributed to the physical changes related to aging.

**Figure 4:**
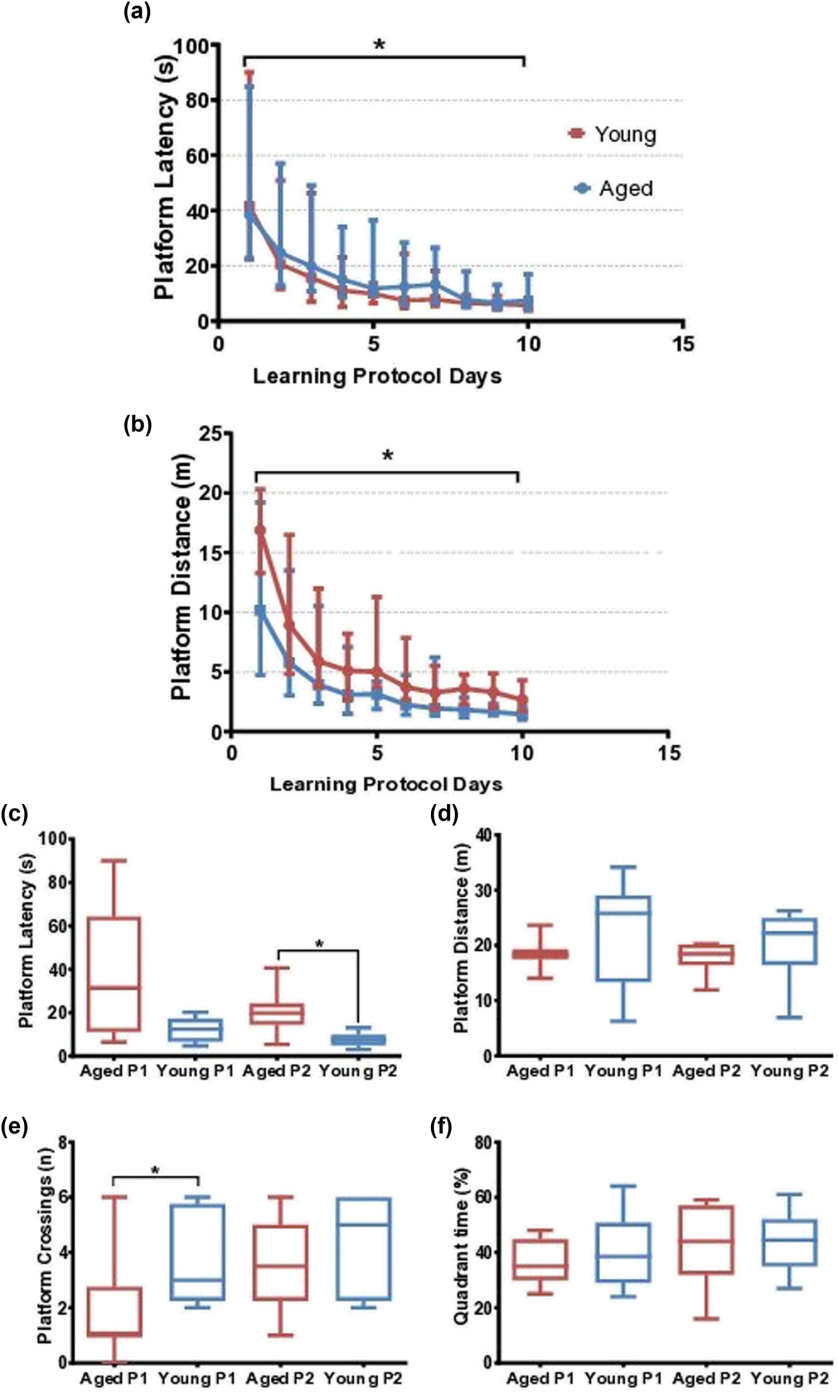
Traditional measurements in the acquisition and probe trials: (a) latency time until the first entry to the platform zone and (b) distance traveled until the first entry to the platform zone during learning days. (c) Latency time until the first entry to the platform zone, (d) distance traveled until the first entry to the platform zone, (e) number of platform crossings, and (f) percentage of time spent in the correct quadrant: probe trials day 1 (P1) and day 2 (P2).

In the acquisition trail, platform latency and distance diminished progressively in both groups showing a significant difference for intragroup comparison using the Friedman test (*p* < 0.0001) (Figure 4a and b). The little distinction between young and aged was identified in the platform latency post hoc analysis where the aged and young groups had statistically significant differences since the third day (aged: day 1 vs days 3 and 5 [*p* <0.05] and day 1 vs days 4, 6–10 [*p* <0.001], while the young: day 1 vs day 3 [*p* <0.05] and day 1 vs days 4–10 [*p* <0.001]) (Figure 4a). In the platform distance post hoc analysis, the little distinction remains (aged: day 1 vs days 3 and 5 [*p* <0.05] and day 1 vs days 4, 6–10 [*p* <0.001], while young: day 1 vs days 4–10 [*p* <0.001] [Figure 4b]).

Regarding the probe trial, statistical differences between aged and young were found in platform latency during the second probe trial (aged 19.8, 14.8–23.6 s vs young 7.6, 5.1–9.1 s, *p* ≤ 0.05 using Mann–Whitney *U* test) and platform crossings during the first probe trial (aged 1, 1–2.5 platform zone crossings vs young 3, 2.5–5.5 platform crossings, *p* ≤ 0.05 using Mann–Whitney *U* test).

Platform distance and quadrant time did not show statistical differences between the aged and young. The distance to reach the platform was similar in both trials and groups (aged rats in P1 [18.3, 17.6–19.1], P2 [18.5, 16.6–20.0], and young rats in P1 [25.8, 15.9–28.4], P2 [22.2, 16.6–24.5]). The percentage of time spent in the correct quadrant was heterogeneous (aged rats in P1 [35, 30–44.5 s], P2 [44, 33–55 s], and young rats in P1 [38.5, 42–47.5 s], P2 [44.5, 37–52 s]).

With these results, we observed that aged rats need a longer acquisition trail to achieve a similar performance than the younger ones, evidenced by the loss of statistical difference between the first and the second memory trials in platform crossings.

### 3.2 Search strategies in the acquisition and probe trials

To standardize the use of the algorithm and avoid interpretation bias, three observers were trained to use the SSA, obtaining an inter-observer Kappa index of 1.0 for efficiency and directionality/localization parameters and a 0.845 Kappa index for precision and classification parameters.

**Figure 5:**
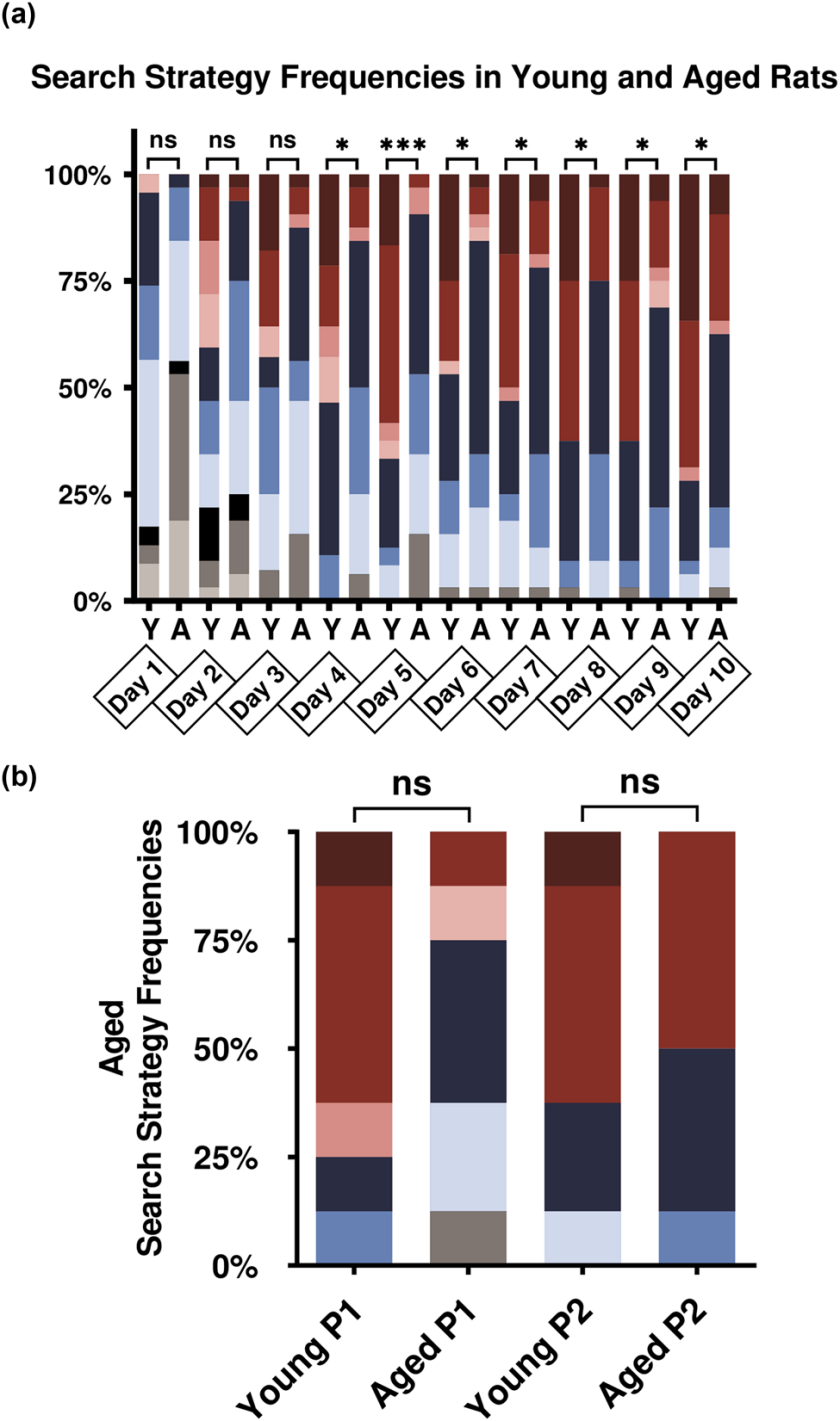
Search strategy changes: (a) acquisition trial day changes in young rats and aged rats; (b) search strategy frequencies in probe trial days P1 and P2.

The pooled group analysis showed that during the acquisition trial, young and aged rats modify their behavior turning from ineffective, indirect, and imprecise to an effective, direct, and precise behavior (Figure 5).

The acquisition protocol demonstrated notable differences between young and aged performance. In the first acquisition trials (days 1–5 in Figure 5a), aged rats developed small changes in their behavior. Young rats showed first a more effective strategy and then used a more localized strategy, refining their strategy and making it more precise at the end of the acquisition protocol on the tenth day. Aged rats showed more difficulties to find the platform, progressing slowly in their effectiveness. On the third acquisition trial, most rats could find the platform and the changes in their strategies to a more precise, however in a slower manner.

In Figure 5, we showed a comparison between the young and aged performance during the acquisition trial days. Chi-Square for qualitative data shows a significant statistical difference in their strategy changes starting on the fourth day and continuing until the tenth (*p* < 0.05), only the fifth day showed a greater statistical significance than the other ones (*p* < 0.0001). These results showed us the gradual process of learning acquisition in both groups, with aged rats using more indirect strategies during the acquisition trail.

The P1 and P2 strategies comparison shown in Figure 5b was made in the memory trials. In this analysis, we can see a predominance of more precise strategies by the young rats while the age rats use predominantly non-localized but effective search strategies. Chi-square show no-differences between both groups in P1 (*p* = 0.117) and P2 trials (*p* = 0.614). The statistical analysis showed differences between young and aged when using the behavioral analysis with the parameter-based algorithm.

## 4 Discussion

As the world population gets old, basic scientific research on aging will be in higher demand. The brain constantly modifies its morphology and function throughout the life span. Therefore, the methods to evaluate its performance should be adapted to better identify this functional decline. The MWM has been used to evaluate the spatial learning and memory process in several situations such as aging, cerebral ischemia, stress, degenerative diseases, and genetic knockout models [21,22,23,24]. We believe that simple and practical methods such as our SSA represents a bridge between highly complex and computerized methods and classical parameters for example traditional measurements in MWM.

### 4.1 Traditional measurements in MWM protocols using aged animals

The initial parameters used by Morris in 1984 were latency, number of crossings to the platform zone, and percentage of time spent in the correct quadrant. The progressive diminishment in these traditional variables indicated aged rats preserve the ability to learn. Aged rats performed longer latencies and distances; however, it was difficult to find a difference in the performance between aged and young rats. Explanations for this are the different velocities between groups, and that variables used in memory probe trials explore the initial search insistence to find the platform, and we observed that during the probe trial aged rats did not insist on the platform zone when they identified the platform was not in the pool. These facts have been observed by others, reporting a high variability in the aged rats’ performance suggesting that aging affects the rat population heterogeneously, complicating the learning analysis [12,25,26]. Gil-Mohapel et al. suggest the differences in platform latency are observed only between very young and aged rodents [10].

We identified three major limitations when using traditional measurements in a normal aging model. First, the difference in latency and distance to the platform may be due to physical changes related to aging (obesity, velocity, and strength) and not necessarily related to cognitive decline. Second, the analysis revealed high variability in the inter-trial and inter-individual results, complicating statistical analysis and interpretation. Third, these parameters do not describe the distribution of time traveling in the pool or the pathway characteristics, producing a lack of descriptive data for the distribution of the search. These limitations justify the need for another measurement of learning and memory, to complement the classic parameters evaluated in MWM, which could be solved by SS analysis.

### 4.2 Search strategy as an indicator of learning and memory

Several authors have proposed SS as a measurement of learning acquisition [10,27]. SS analysis describes the process of searching, providing a detailed description of the process of learning and memory acquisition. During the experiment, we observed a different behavior to reach the platform. While some rats performed direct trajectories to reach the platform with little or any changes, other rats performed indirect trajectories, like seeking more external cues. Some authors associated these different behaviors with allocentric (direct/spatial) and egocentric (indirect/non-spatial) learning [25].

Navigation through the space is a complex process in which the animals use different strategies to arrive at some point (i.e., their nest). The strategies can involve the use of signals that could be far away or near them (allocentric) or route-based space navigation, where the animal follows a sequence of movements to find the objective point (egocentric). Hippocampal areas are key in the development and use of allocentric strategies, while egocentric strategies are less studied, but the entorhinal cortex, striatum, and thalamus are involved [8,28].

### 4.3 Learning and memory differences in aged rats

Our study demonstrated a different behavior between aged and young rats during the acquisition trial. An increasing number of young rats increased the number of effective search strategies; then, the number of effective and direct strategies, and in the last days, improves the precision. In contrast, aged rats needed more days of training to obtain an effective strategy, even though they improved their strategies increasing the effectiveness with indirect strategies (blue colors), only a few aged rats improve precision. Probably with a greater extension of the acquisition trial, aged rats would acquire direct search strategies as the younger.

Learning and memory abilities remain relatively constant along with the lifespan of the rodent [10,13]. Two main cognitive performances can be used to find the platform: spatial and non-spatial [28,29]. The use of different search strategies has been reported along different stages of life in rodents [10,12,25]. While young rats used spatial-based SS (allocentric), aged rats used indirect, non-spatial SS (egocentric) [17]. This means that spatial learning acquisition abilities decline with age [25]. The acquisition of imprecise/indirect SS has been correlated with the reduction in hippocampal neurogenesis [10]; also, an increase of neurogenesis in the dentate gyrus provoked by the deep brain stimulation enhanced MWM performance in mice [30]. Acquired defects may also deteriorate spatial memory performance as occurs after traumatic brain injury [20]. Although the normal aging process is a less studied field, evidence has shown that the decrease in spatial learning abilities could be related to decreased levels of hippocampal neurogenesis [31].

Our evidence indicates that aged rats had a restricted spatial learning capacity. This is reflected in indirect, non-localized strategies used in most of the experiments and decreased performance in the aged rats in the learning and probe trials. These results suggest that normal aging modifies the use of the spatial abilities reflected in indirect and non-spatial trajectories.

### 4.4 Advantages and pitfalls with existing methods

Several methods have been proposed to evaluate SS in MWM [10,27,32,33]; most of them used computer programs, algorithms, or qualitative analysis. Computerized programs use complicated methods difficult to reproduce by young research groups. Different algorithms and classifications have been purposed. The algorithms created by different authors normally consider the effectivity and directionality [27] but the precision (accuracy) is a more controversial parameter, used in different ways through the SS analysis [34]. These three major parameters (efficiency, directionality, and precision) were used to design the SSA.

The proposed SSA (Figure 2) is an optional methodology to analyze the process of learning and memory acquisition, allowing good reproducibility, data interpretation, and solving the methodological problem of the variability in the traditional measurements. The parameters used in SSA allow us to classify the direct and indirect spatial searching trajectories into subclassifications based on the initial direction and trajectory changes, establishing a hierarchical order which could be useful to evaluate subtle changes in the process of learning.

Its advantages are (1) the absence of unclassified search strategies, (2) easy reproducibility with two observers, and a third to decide the discrepant results, (3) sophisticated and computerized programs are not needed, (4) differentiation of spatial and non-spatial SS, (5) parameter-based decision-making algorithm, (6) new alternative for the non-computerized evaluation of the SS, (7) it is not modified by physical aspects such as velocity and swimming abilities, and (8) it represents a more sensible to evaluate subtle changes in the learning process. However, SSA has some limitations, like not considering if the sequence of movements changes through different trials or the left and right distinction. Another limitation to guaranteeing a good reproducibility is that it must be performed by two independent observers blinded to the group distribution meaning an increase in the time of evaluation.

With these aspects in mind, SSA could be used in MWM research to better explore spatial learning and memory deficits, as occurs during normal aging, structural damage, or global cerebral ischemia. Alternative measurements are path directionality or cumulative distance to the platform, which may allow a more detailed distinction between patterns of SS although these measurements do not examine the entire trial. The velocity and swimming abilities were different, most likely due to differences in body weight, physical development, and age [13], distorting platform latency and assessment of distance traveled.

## 5 Conclusion

As the global population continues to get older, age-related diseases are more prevalent, hence the need for adequate animal models that consider aging research. Adaptations in the assessment tools and methods such as MWM are needed to better examine and understand the aging process and the response of the aged brain to pathological phenomena. The MWM is a common and accepted method to assess spatial learning and memory; unfortunately, the high variability in the performance of aged rats in this test complicates the assessment, interpretation, and comparison of the results. Our study designed an algorithm that effectively evaluated these variables, creating a model to assess learning and memory impairment. Additionally, we conclude that SS is a suitable variable to assess learning and memory in MWM protocols in aged and young rats. The proposed SSA is a reliable and reproducible method to analyze and classify search strategies during MWM protocols.
